# Genome sequence and pathogenicity of *Vibrio vulnificus* strain MCCC 1A08743 isolated from contaminated prawns

**DOI:** 10.1242/bio.059299

**Published:** 2022-06-29

**Authors:** Jie Li, Yiqing Zhu, Zhenxia Ma, Fu Yang

**Affiliations:** 1 Department of Medical Genetics, Naval Medical University, Shanghai 200433, China; 2 Department of Biochemistry and Molecular Biology, Naval Medical University, Shanghai, 200433, China

**Keywords:** *Vibrio vulnificus*, Genome sequencing, Pathogenicity, Core genome, Liver lesions

## Abstract

*Vibrio vulnificus* is an opportunistic pathogen that naturally inhabits sea water globally and is responsible for most vibriosis-related deaths. The consumption of *V. vulnificus* contaminated seafood and exposure of wounds to *Vibrio* can result in systemic infection, with increased risks of amputation and extremely high rates of mortality. However, the pathogenicity and virulence factors of *V. vulnificus* are not fully understood. The genomic characterization of *V. vulnificus* will be helpful to extend our understanding on *V. vulnificus* at a genomic level. In this manuscript, the genome of *V. vulnificus* strain MCCC 1A08743 isolated from contaminated prawns from Zhanjiang, China, was sequenced using Illumina HiSeq X Ten system and annotated through multiple databases. The strain MCCC 1A08743 genome included 4371 protein-coding genes and 117 RNA genes. Average nucleotide identity analysis and core genome phylogenetic analysis revealed that MCCC 1A08743 was most closely related to strains from clinical samples from the United States. Pathogenicity annotation of the MCCC 1A08743 genome, using Virulence Factor Database and Pathogen-Host Interactions database, predicted the pathogenicity of the strain, and this was confirmed using mice infection experiments, which indicated that *V. vulnificus* strain MCCC 1A08743 could infect C57BL/6J mice and cause liver lesions.

This article has an associated First Person interview with the first author of the paper.

## Abstract (precis)

**Summary:** *Vibrio vulnificus* strain MCCC 1A08743 was newly isolated, sequenced and tested for its pathogenicity in mice.

## INTRODUCTION

*Vibrio vulnificus* is a gram-negative bacillus that is responsible for most of the world's vibriosis-related deaths. *Vibrio vulnificus* is usually found in warm, shallow, coastal salt water in temperate climates, and people generally become infected through either the consumption of *V*. *vulnificus*-contaminated seafood or the exposure of open wound. In the United States, *V. vulnificus* is the leading cause of seafood consumption-related deaths, and vibriosis is reported to cause ∼80,000 illnesses and ∼100 deaths each year (www.cdc.gov/vibrio/index.html). Some patients experience gastroenteritis, whereas others may suffer from severe forms of infection, such as sepsis, severe cellulitis, and necrotising fasciitis (Jones and Oliver, 2009). Patients with pre-existing medical conditions, such as chronic liver disease, diabetes mellitus, hemochromatosis, and thalassemia major, are more likely to incur life-threatening forms of infection (Oliver, 2006).

A complete genomic picture is needed to understand the mechanisms underlying *V. vulnificus* pathogenicity and evolution more thoroughly, and to explore strategies for decreasing *V. vulnificus* loads in marine animals and reducing incidence of invasive disease. However, there are currently 238 genome assembly and annotation reports of *V. vulnificus* available in NCBI databases, and more than half of the reports are strains from the United States, with less than 10% of strains reported from mainland China. Furthermore, *V. vulnificus*-related amputation and death have increased in China, with mortality rates ranging from 18 to 56% (Chao et al., 2013; Hong et al., 2014).

Accordingly, the aims of the present study were to sequence and annotate the genome of *V. vulnificus* strain MCCC 1A08743, which was isolated from contaminated prawns without evidence of disease in Zhanjiang, China, and to verify the strain's pathogenicity using animal experiments. The results of these experiments indicated that MCCC 1A08743 held the potential for infection and clustered with clinically isolated *V*. *vulnificus* strains from the United States, and this finding was confirmed by the lethality of the strain in mice infection experiments.

## RESULTS

### General genomic features of strain MCCC 1A08743

An Illumina HiSeq paired-ended sequencing run resulted in 7,944,730 reads with a total read length of 1,197,306,181 bp. The assembled genome had depth of 200× coverage (N50=175,749 bp, largest scaffold=428,496 bp, scaffold number=83). The genome of MCCC 1A08743 has a size of 4,825,020 bp with a mean G+C content of 46.70%. A total of 4371 protein coding genes and 117 RNA genes were predicted, and the general features of strain MCCC 1A08743 are summarised (Tables 1–3).

**Table 1.**
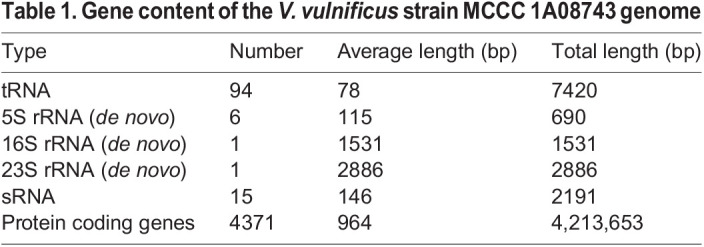
Gene content of the *V. vulnificus* strain MCCC 1A08743 genome

**Table 2.**
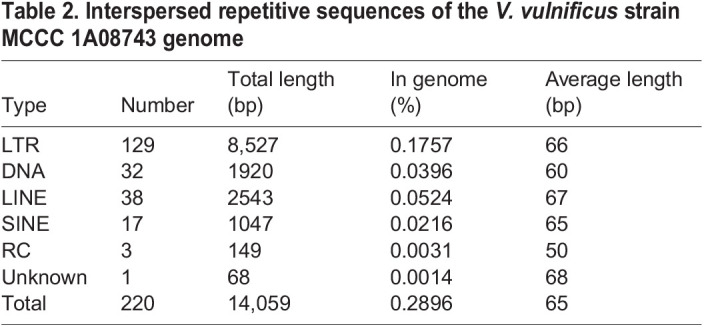
Interspersed repetitive sequences of the *V. vulnificus* strain MCCC 1A08743 genome

**Table 3.**
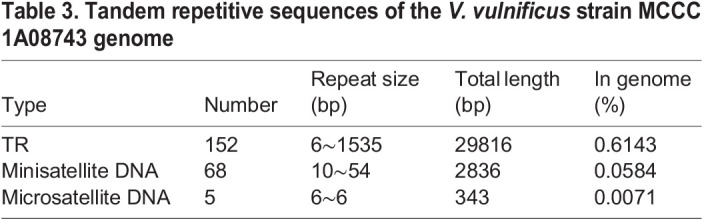
Tandem repetitive sequences of the *V. vulnificus* strain MCCC 1A08743 genome

### Functional annotation of the predicted genes of strain MCCC 1A08743

**Fig. 1.**
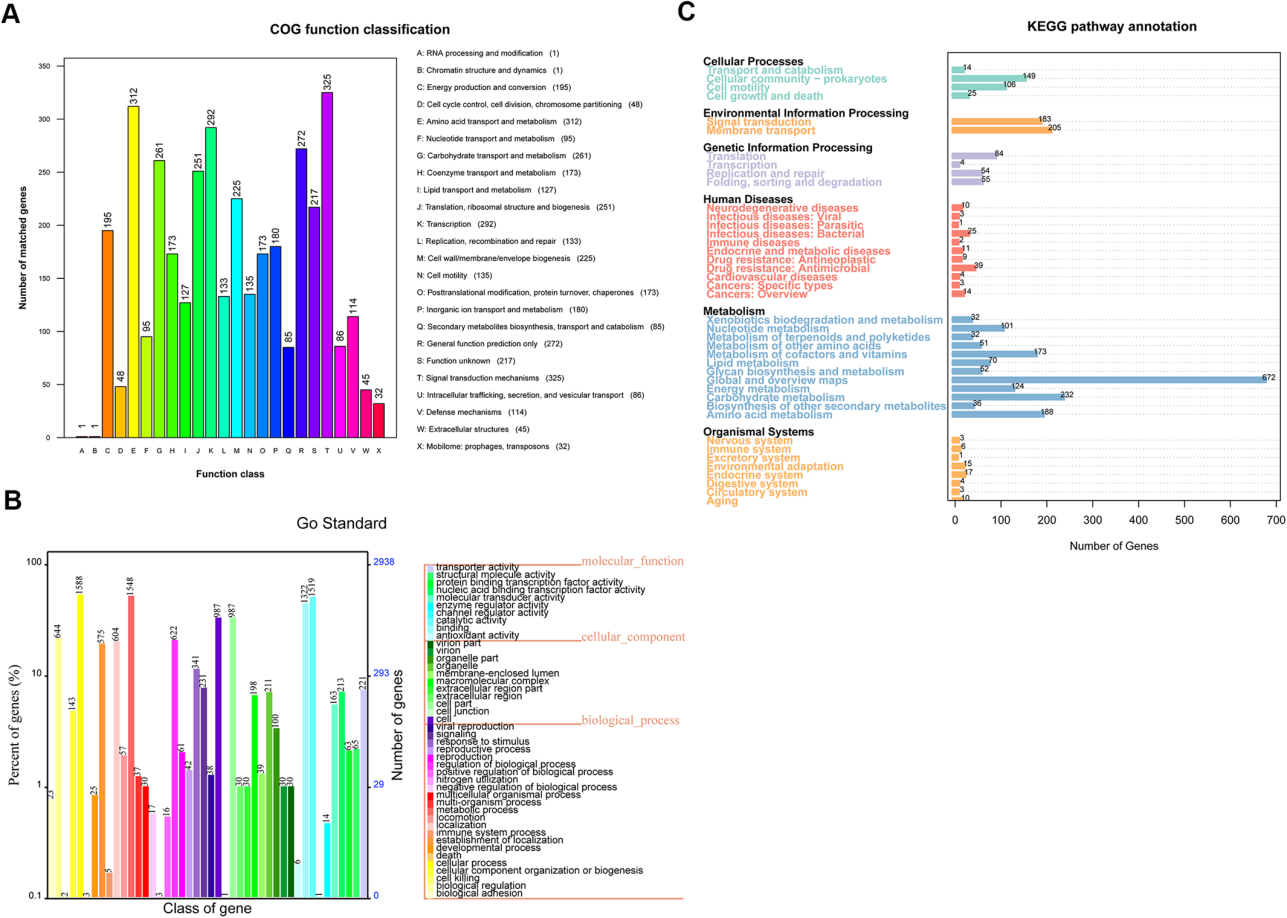
**Functional annotation of the genome of *V. vulnificus* strain MCCC 1A08743.** (A) COG functional classification of the genome of *V. vulnificus* strain MCCC 1A08743. (B) GO annotation of the genome of *V. vulnificus* strain MCCC 1A08743. (C) KEGG annotation of the genome of *V. vulnificus* strain MCCC 1A08743.

The putative functions of most of the protein-coding genes were prognosticated through multiple databases, and the gene annotation of strain MCCC 1A08743 was summarised (Table S1). The distribution of these genes into COGs (clusters of orthologous groups) functional categories was analysed, among which, signal transduction mechanisms ranked the largest category with 325 genes (Fig. 1A). The molecular functions, cellular components and biological processes of these genes were elucidated by GO (Gene Ontology) terms, and the largest two categories of strain MCCC 1A08743 annotated genes were assigned to cellular process (1588 genes) and metabolic process (1548 genes) (Fig. 1B). Furthermore, the signal processes of these genes were described by KEGG (Kyoto Encyclopaedia of Genes and Genomes) pathways, most of the annotated genes were involved in metabolism, especially the metabolic pathways belonging to global and overview maps (672 genes) (Fig. 1C). Annotation results using TCDB (Transporter Classification Database) and CAZy (Carbohydrate-Active Enzyme database) were shown in the supplementary files (Figs S1 and S2).

### Core genome analysis and phylogenetic analysis

**Fig. 2.**
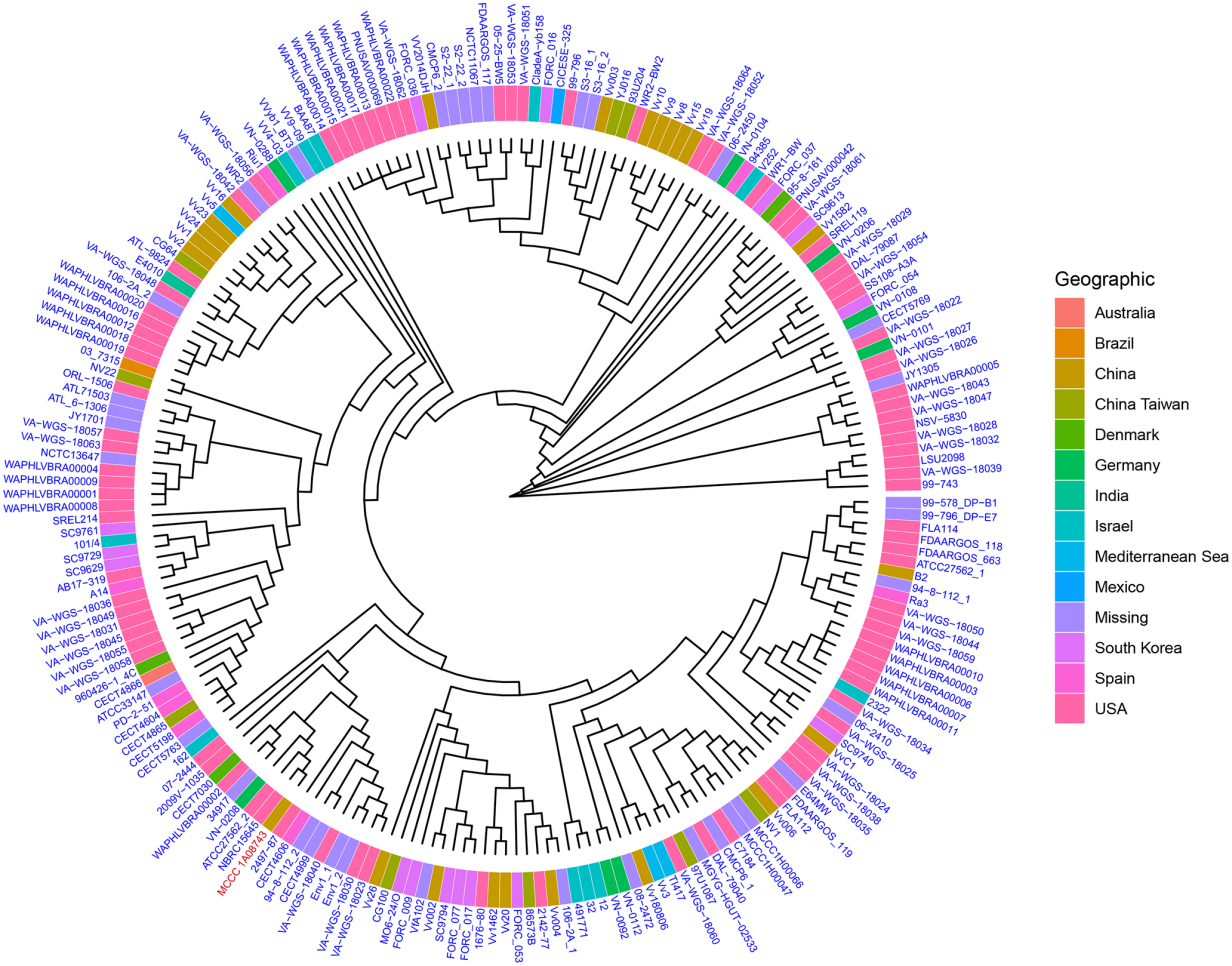
**Phylogenetic tree of *V. vulnificus* core genome sequences.** *Vibrio vulnificus* strain MCCC 1A08743 is highlighted in red.

Among the 212 *V. vulnificus* strains analysed, the core and pan genome size were 2248 and 28,290, respectively. The 212 strains of *V. vulnificus* were distributed into distinct clades of a phylogenetic tree based on the core genome (2248 genes) of these strains, which suggested that global *V. vulnificus* is likely to be heterogeneous. Interestingly, strain MCCC 1A08743 was assigned to a specific branch with the strain NBRC15645. According to the NCBI database, NBRC15645 is equal to ATCC 27562, which was isolated from a clinical sample in the United States. Both strains were clustered with another two strains (ATCC 27562_2 and VN-0206), among which, ATCC 27562_2 represents the genome sequence of ATCC 27562 with contig level assembly completed in the year 2016, while VN-0206 was isolated from a German environmental sample (Fig. 2). Even though strain MCCC 1A08743 was isolated from prawns, it appears to be most similar to a strain isolated from a clinical sample in the US. This phylogenetic analysis suggested that strain MCCC 1A08743 could possess biological properties that are like those of strains from clinical samples in the United States.

### Average nucleotide identity analysis

**Fig. 3.**
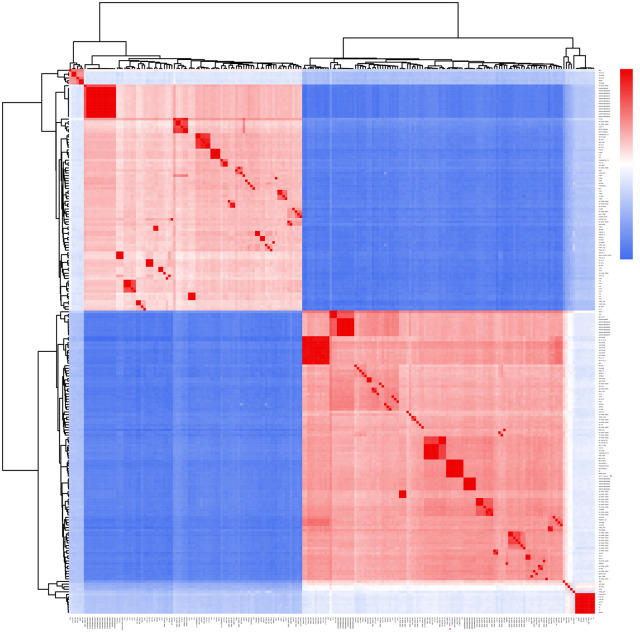
**Heat map of ANI of 212 *V. vulnificus* strains.** Blue colour represents low identity and red colour represents high identity. *Vibrio vulnificus* strain MCCC 1A08743 is highlighted in purple with red asterisk.

ANI (average nucleotide identity) analysis was conducted with the full-length genome sequence of strain MCCC 1A08743 and 211 other *V. vulnificus* strains, in order to measure the nucleotide-level genomic similarity between them (Fig. 3; Table S2). ANI varied from 95.04–100% among the 212 worldwide strains of *V. vulnificus*. Strain MCCC 1A08743 from China had a 95.30–99.99% ANI with the 211 other strains of *V. vulnificus* in the NCBI database. Strain MCCC 1A08743 harboured the highest identity (99.99%) with strain ATCC 27562_2, ATCC 27562_1, NBRC15645, and FDAARGOS_663, all of which were isolated from the United States clinical samples. ATCC 27562_1 represents the genome sequence of ATCC 27562 with complete level assembly completed in the year 2017. In addition, strain MCCC 1A08743 had the second highest identity (99.98%) with strain B2, isolated from human blood sample in China. Thus, the ANI analysis and phylogenetic analysis based on core genome reach the same conclusion, strain MCCC 1A08743 might have more similarities with clinically isolated *V. vulnificus* strains.

### Pathogenicity prediction of strain MCCC 1A08743

**Fig. 4.**
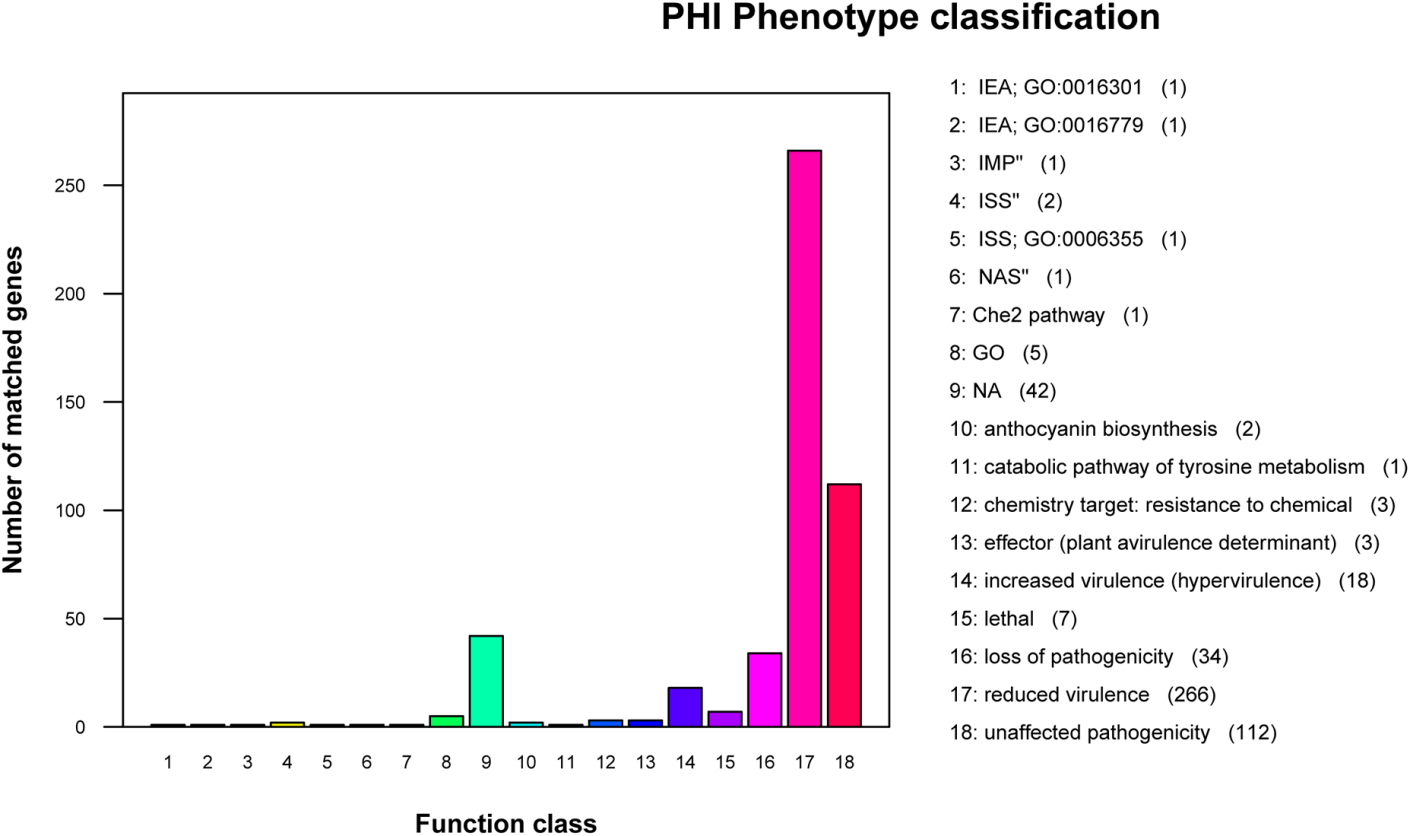
**Phenotype classification of putative pathogenicity genes in *V. vulnificus* MCCC 1A08743 genome.** Information is based on data retrieved from PHI-base.

Several virulence factors of *V. vulnificus* contribute to its pathogenicity have been reported, such as hemolysin, VvpE (Fan et al., 2001; Jones and Oliver, 2009), RtxA1 toxin (Chung et al., 2010; Kim et al., 2008; Lee et al., 2007), the capsular polysaccharide (CPS), lipopolysaccharide (LPS) (Ellis and Kuehn, 2010; Vanaja et al., 2016), flagellum, and pili (Jenne and Kubes, 2013; Lee et al., 2004). Thus, we searched the VFDB database (Virulence Factor Database) with our genome sequence to annotate the virulence factors of the strain MCCC 1A08743. The results showed that 388 genes were annotated, and most of the canonical virulence factors were found in the genome of strain MCCC 1A08743, including 64 flagella-related genes, 38 genes related to pili, and eight genes related to different secretion systems, six genes related to RTX toxin, four genes related to hemolysin, eight genes related to LPS, and 19 genes related to CPS (Table S3). We further analysed the pathogenicity of strain MCCC 1A08743 through PHI base (the Pathogen-Host Interactions database), 501 genes were annotated, and the pathogen-host interaction phenotype classification was summarised in Fig. 4. According to the PHI annotation, 266 of 501 annotated genes were classified into ‘reduced virulence’ phenotypic outcome term, 34 genes mutation potentially led to loss of pathogenicity.

### Acute infection by *V. vulnificus* strain MCCC 1A08743 induced liver lesions in C57BL/6J mice

**Fig. 5.**
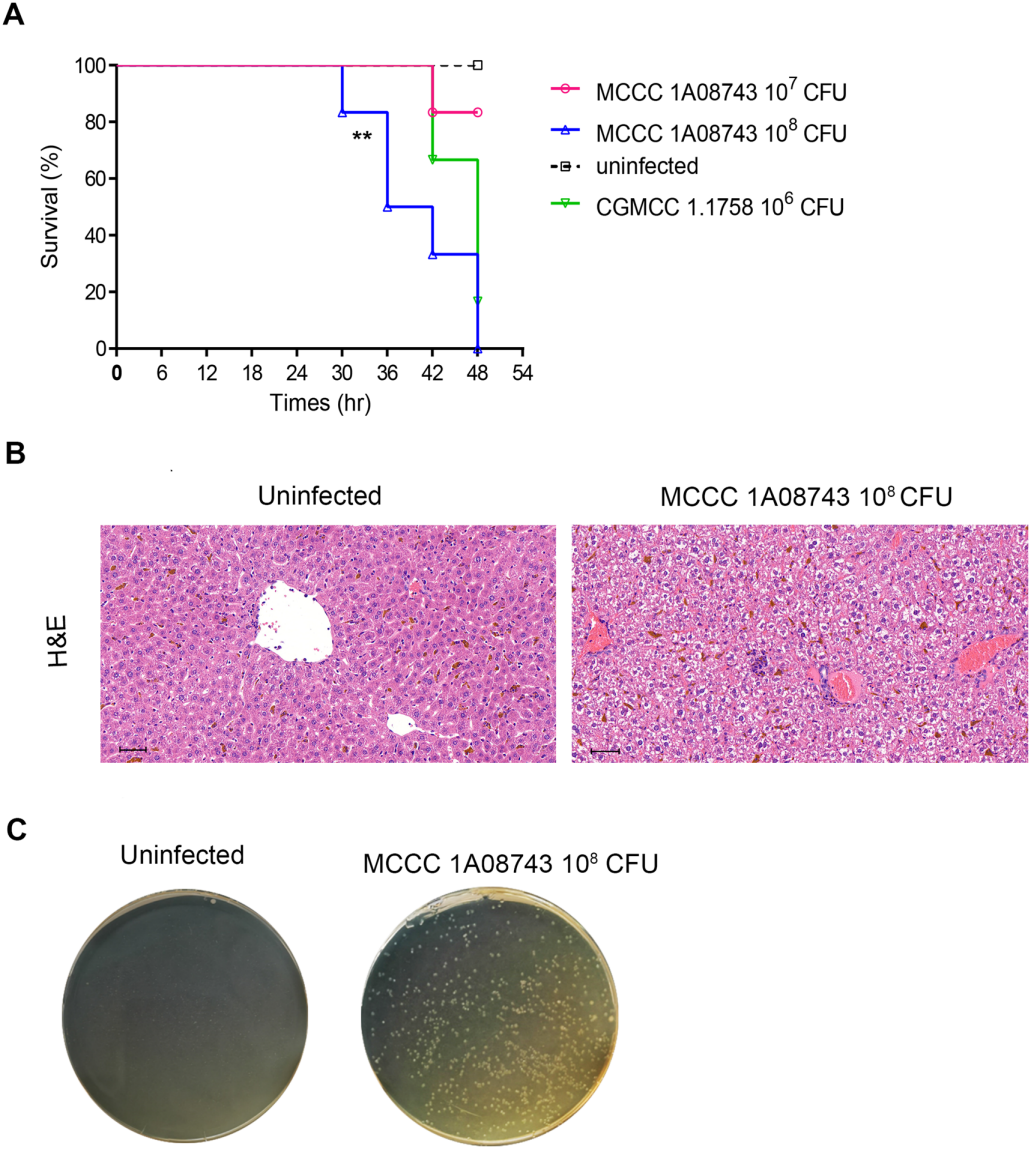
**Induction of liver lesions by *V. vulnificus* MCCC 1A08743 strain acute infection.** (A) Survival curves for 6- to 8-week-old C57BL/6J mice that received subcutaneous injections of 1×10^7^ or 1×10^8^ CFU *V. vulnificus* strain MCCC 1A08743, or of 1×10^6^ CFU *V. vulnificus* strain CGMCC 1.1758 (positive control). Asterisks (**) indicate a significant difference (**P<0.01) in the survival of uninfected and infected (1×10^8^ CFU *V. vulnificus* strain MCCC 1A08743) mice assessed with the log-rank survival analysis (*n*=6 in each group). (B) Haematoxylin and Eosin-stained liver sections prepared from uninfected and infected (1×10^8^ CFU *V. vulnificus* strain MCCC 1A08743) mice 12 h after injection. Partial hepatocytes show brown iron deposits. Original magnification: ×200. (C) Detection of *V. vulnificus* after overnight culture of liver homogenates on BHI agar plates. Liver homogenates were prepared from samples collected 12 h post 1×10^8^ CFU MCCC 1A08743 infection. Data shown are representative of at least two experiments.

Because several other *V. vulnificus* strains have been reported to cause systemic infection and even mortality in mice (Jeong and Satchell, 2012; Kashimoto et al., 2003; Liu et al., 2015; Starks et al., 2000), the present study investigated whether strain MCCC 1A08743 would be lethal to mice. Mice subcutaneously injected with 1×10^8^ CFU strain MCCC 1A08743 exhibited a mortality rate that was higher than that of mice injected with 1×10^7^ CFU strain MCCC 1A08743, but similar to that of mice injected with the positive control (1×10^6^ CFU strain CGMCC 1.1758) (Fig. 5A). This indicates that the strain MCCC 1A08743 had to be inoculated at a dose of approximately 100-fold higher than that of the clinical model strain CGMCC 1.1758 in order to be able to cause an equivalent frequency and magnitude of infection. According to previous research, most environmental strains including those isolated from marine animals exhibited attenuated pathogenic process about 350- to 1500-fold higher than clinical strains (Starks et al., 2000). Thus, strain MCCC 1A08743 seems to possess a higher infection capacity than the majority of environmental *V. vulnificus* strains.

Patients with liver disease are susceptible to severe *V. vulnificus* infections. One of the key functions of the liver is to remove blood-borne pathogens and to prevent the spread of bacteria to other organs and cause sepsis (Jenne and Kubes, 2013). A recent study reported that *V. vulnificus* caused liver inflammation in mice (Xie et al., 2017). To evaluate whether *V. vulnificus* strain MCCC 1A08743 could also cause liver lesions, C57BL/6J mice were subcutaneously injected with strain MCCC 1A08743. Mice injected with 10^8^ CFU MCCC 1A08743 exhibited mortality in 48 h, and Haematoxylin and Eosin staining revealed that MCCC 1A08743 also caused liver lesions within 12 h of infection, as indicated by hepatic sinusoid hyperaemia, loss of normal hepatic architecture and piecemeal necrosis (Fig. 5B). Strain MCCC 1A08743 was also detected in the liver homogenates of infected mice (Fig. 5C). These observations indicate that *V. vulnificus* strain MCCC 1A08743 is capable of inducing liver lesions in C57BL/6J mice. Thus, *in vivo* infection experiments in mice confirm that the strain MCCC 1A08743 is pathogenic, which is consistent with the results of genomic bioinformatics analysis.

## DISCUSSION

In recent years, *V. vulnificus* infection cases have been reported with increasing frequency in China. Currently, treatment methods for *V. vulnificus* mainly include antibiotics, aggressive wound therapy, and other supportive care. Prompt diagnosis and aggressive treatment will significantly improve patient outcomes (Bross et al., 2007). It is well known that genome analysis is useful for bacterial taxonomy, pathogenicity prediction, antimicrobial resistance prediction and so on. While, among the 238 *V. vulnificus* genome information in GenBank with complete or scaffold sequences globally, only ten genomes were reported in mainland China. In this study, the whole genome of *V. vulnificus* strain MCCC 1A08743, which was isolated from contaminated prawns from Zhanjiang, China, was sequenced, assembled, and annotated using multiple databases. The annotations performed with multiple databases including GO, COG, KEGG, TCDB and CAZy, and most of the functional genes were involved in the cellular process (1588 genes) and metabolic process (1548 genes).

By comparing the ANI value from strain MCCC 1A08743 and 211 other strains of *V. vulnificus* worldwide, we found that strain MCCC 1A08743 was most similar (99.99%) to strains derived from US clinical samples, which was consistent with the results of core genome comparison and phylogenetic analysis, and together, these results indicate that strain MCCC 1A08743 could harbour biological properties that are similar to those of strains from United States clinical samples. Although the *V. vulnificus* genes and processes leading to human diseases are not well understood, several canonical virulence factors of *V. vulnificus* either contributing to growth in serum, inflammation, immune evasion or invasion could be found in the genome of the strain MCCC 1A08743 (Carda-Diéguez et al., 2018) such as hemolysin, VvpE (Fan et al., 2001; Jones and Oliver, 2009), RtxA1 toxin (Chung et al., 2010; Kim et al., 2008; Lee et al., 2007), the CPS, LPS (Ellis and Kuehn, 2010; Vanaja et al., 2016), flagellum, and pili (Jenne and Kubes, 2013; Lee et al., 2004). This was done by pathogenicity prediction analysis through VFDB databases. In addition, some other virulence factors were also predicted and summarised in Table S3, which will serve as a reference for future research.

The PHI-base catalogues pathogenicity, virulence and effector genes from pathogens verified by experiments (Urban et al., 2015). According to the PHI annotation, 266 of 501 annotated genes were classified into ‘reduced virulence’ phenotypic outcome term, including housekeeping genes (mdh, pyrC), pathogenic genes (VvpE, Flagellin, pilA). 34 genes mutation potentially led to loss of pathogenicity, such as murF, murD, BdlA, Grx4, YbeY, the function and characteristics of which were less reported in *V. vulnificus*.

All the above information from genome analysis indicates the strain MCCC 1A08743 are infectious. Finally, an *in vivo* experiment was conducted, using C57BL/6J mice to confirm the pathogenicity of strain MCCC 1A08743. In order to obtain a more pronounced liver phenotype, we tested the lethal dose of *V. vulnificus* MCCC 1A08743 and CGMCC 1.1758 in our laboratory condition, which is consistent with other researchers (Jeong and Satchell, 2012; Kwak et al., 2011). The Kaplan-Meier survival curve of mice subcutaneously infected with MCCC 1A08743 at a dose of 1×10^8^ CFU was significantly different from that of the uninfected group, but not significantly different from that of clinical model strain CGMCC 1.1758 (1×10^6^ CFU). According to reports from the Centers for Disease Control and Prevention (CDC), patients with chronic liver disease or immunocompromising conditions are susceptible to *V. vulnificus* infection with a more severe phenotype (CDC, 2020). In this study, we confirmed that MCCC 1A08743 itself could lead to liver lesions in normal mouse model. Report showed that *V. vulnificus rtxA* mutant reduced the spread of *V. vulnificus* to the liver, which also indicated the function of toxin in the development of systemic disease (Gulig et al., 2005). Another virulence gene *vvhA* was associated with the lysis of various cell types, such as erythrocytes, epithelial cells and macrophages through different molecular mechanisms (Jeong and Satchell, 2012). However, the way by which the *V. vulnificus* virulence genes associated with liver infection and liver lesions needs more exploration. In addition, the results of MCCC 1A08743 infection experiments in non-alcoholic fatty liver disease (NAFLD) mouse model also showed a more severe phenotype than normal mouse model (data not shown), and we are exploring the mechanisms behind it.

In conclusion, this study improves the current genome-level understanding of *V. vulnificus* and may provide useful information for future epidemiological investigation and research of pathogenicity and virulence factors.

## MATERIALS AND METHODS

### Genomic DNA isolation

A single colony of MCCC 1A08743 was inoculated into 5 ml brain heart infusion broth (BHI) and cultured at 28°C with vigorous shaking (180 rpm). Genomic DNA was isolated from 3 ml culture broth using an UltraPure™ DNA extraction kit (SBS Genetech, Beijing, China) and the quality of the purified genomic DNA was evaluated using a NanoDrop 2000 spectrophotometer (Thermo Fisher Scientific, MA, USA) and a Qubit 2.0 fluorometer (Life Technologies, MA, USA). *Vibrio vulnificus* strain MCCC 1A08743 was purchased from the Marine Culture Collection of China (MCCC, Xiamen, China), with deposited number: MCCC 1A08743.

### Genome sequencing and assembly

The genome of MCCC 1A08743 was sequenced using the Illumina HiSeq X Ten system with the strategy of 150 paired-ends reading at Shanghai Majorbio Bio-Pharm Technology Co., Ltd. (Shanghai, China). About 1.1 gigabytes of clean data were obtained with over 200× coverage after using readfq (version 10, https://github.com/lh3/readfq) to remove adapter sequences and sequences with either low-quality bases or high N-percentages. SOAPdenovo 2.04 (Li et al., 2008, 2010), SPAdes (Bankevich et al., 2012) and ABySS (Simpson et al., 2009) were utilised to perform *de novo* genome assembly, and the assemblies were integrated into a hybrid set of contigs using CISA software (Lin and Liao, 2013). GapCloser v1.12 was used to optimise the preliminary assembly results to obtain the final assembly results. Small fragments (<500 bp) were filtered out and the contaminated samples were decontaminated again to prepare for subsequent gene prediction.

### Genome annotation and analysis

Protein-coding genes were predicted using GeneMarkS version 4.17 (http://topaz.gatech.edu/GeneMark/) (Besemer et al., 2001). Repetitive and low-complexity regions of the scaffolds were identified using RepeatMasker (Version open-4.0.5) (Saha et al., 2008), and TRF (Tandem Repeats Finder, Version 4.07b) (Benson, 1999) was used to identify tandem repeats. Transfer RNAs (tRNAs) were predicted using tRNAscan-SE software (Version 1.3.1) (Lowe and Eddy, 1997), and ribosomal RNAs (rRNAs) were predicted using RNAmmer software (Version 1.2) (Lagesen et al., 2007). In addition, small RNAs (sRNAs) were compared using the Rfam database (https://rfam.xfam.org/), and then confirmed by the CMsearch program (Version 1.1rc4) with default parameters. Gene annotations were determined with the BLASTP program (E-value <1×10^−5^, identity ≥40%, coverage ≥40%) and six databases including COG (Tatusov et al., 2003), GO (Ashburner et al., 2000), KEGG (Kanehisa et al., 2004, 2006), TCDB (Saier et al., 2016), CAZy (Cantarel et al., 2009) and Swiss-Prot (Bairoch and Apweiler, 2000).

Furthermore, since *V. vulnificus* strain MCCC 1A08743 is a pathogenic bacterium, pathogenicity and drug resistance data were retrieved from PHI-base (Urban et al., 2015), and virulence factors were predicted using VFDB (Chen et al., 2012).

### Core genome analysis

A total of 211 of the 238 *V. vulnificus* genome sequences available in NCBI were selected upon review of both the cluster size distribution and the number of unique clusters across the set of all strains (Table S4). Core genes and specific genes of the 211 strains of *V. vulnificus* and MCCC 1A08743 were analysed using the CD-HIT (V4.6) fast clustering of similar protein sequence with default parameters (Li and Godzik, 2006). We determined the core genome cut-off through sensitivity analysis by maximizing the second derivative of the curve for the relationship between core genome genes number and the number of strains. The core genome profile trend represents the change in core genome to changes in the number of strains a gene must be found in to be defined as core (Fig. S3) (Kavvas et al., 2018).

### Phylogenetic analysis and ANI

We created a phylogenetic tree of 212 strains including newly sequenced strain MCCC 1A08743 and the other 211 selected *V. vulnificus* strains. Specifically, we chose a set of 2248 core genes through core genome analysis. The gene family clustering was based on the multiple sequence alignment results using MUSCLE software (Edgar, 2004). A maximum likelihood phylogenetic tree, based on 2248 core genes conserved across the 212 strains of *V. vulnificus*, was constructed using FastTree software (Price et al., 2010), and the phylogenetic tree was visualised with ITOL software (Letunic and Bork, 2007). The ANI between MCCC 1A08743 and the other 211 strains were calculated by fastANI with the default parameters (Jain et al., 2018).

### Direct link to deposited data and information to users

The complete genome sequence of MCCC 1A08743 has been deposited in GenBank under the accession number JAILXG000000000.

### Infection of mice with *V. vulnificus* MCCC 1A08743

Six- to eight-week-old specific pathogen-free male C57BL/6J mice weighing approximately 20 g were purchased from Shanghai SLAC Laboratory Animal Co., Ltd. (Shanghai, China). The mice were pre-treated with iron dextran at 1 h before treatment as previously described (Starks et al., 2000), and then subcutaneously injected with either 0.1 ml *V. vulnificus* MCCC 1A08743 (∼10^9^ CFU/ml or 10^8^ CFU/ml), 0.1 ml *V. vulnificus* CGMCC 1.1758 (∼10^7^ CFU/ml; positive control), or phosphate-buffered saline (PBS) with 0.01% (wt/vol) gelatine (negative control).

The number of dead mice within 48 h and their survival time were recorded. Six mice were used for each dose, and the experiment was repeated twice. To measure the *V*. *vulnificus* colony forming units (CFUs) of the mouse livers, infected mice were perfused with PBS, and then the livers were harvested, homogenised in PBS, diluted, and further cultured on BHI agar plates at 28°C for 24 h. For biosecurity, the infected mice were disposed by autoclaving at the end of the experiments. All animal experiments were approved by the Committee on Ethics of Medicine, Naval Medical University, PLA.

*Vibrio vulnificus* strain CGMCC 1.1758 was purchased from the Marine Culture Collection of China (MCCC, Xiamen, China), with deposited number: CGMCC 1.1758. The original number of CGMCC 1.1758 is ATCC 27562.

### Histological analysis

Specimens of liver were collected immediately after sacrifice and fixed in 4% paraformaldehyde solution (PFA). The fixed tissues were then embedded in paraffin and cut into thin sections (5–10 µm). Histological sections were routinely stained with Haematoxylin and Eosin.

### Statistical analysis

All statistical analyses were performed using GraphPad Prism 5.0 software. For comparisons, log-rank test was performed as indicated in figure legends to compare survival curves. A *P*-value <0.05 was considered significant.

## Ethics approval and consent to participate

All animal experiments were approved by the Committee on Ethics of Medicine, Naval Medical University, PLA. This study was carried out in accordance with relevant institutional guidelines and regulations. All animal experiments were carried out in compliance with the ARRIVE guidelines.

## Supplementary Material

Supplementary information
