# Supplementary material for: Genome sequence and pathogenicity of Vibrio vulnificus strain MCCC 1A08743 isolated from contaminated prawns
Source: Biol Open. 2022 Jun 29;11(6):bio059299. doi: 10.1242/bio.059299 (PMC9253834; doi:10.1242/bio.059299)
Supplement: Supplementary information [file biolopen-11-059299-s1.pdf]

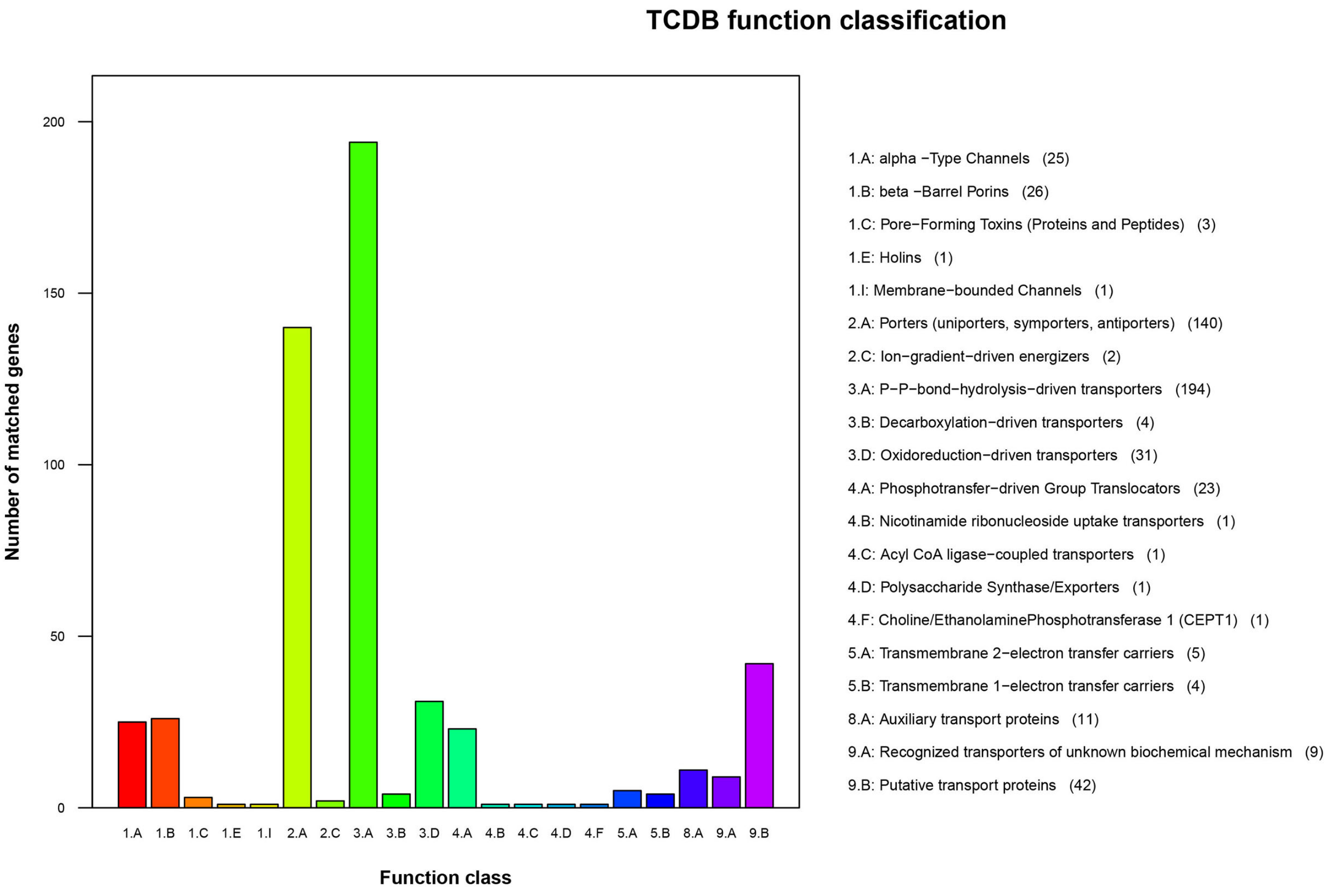

Fig. S1. TCDB annotation of the protein coding sequences of the genome of *V. vulnificus* strain MCCC 1A08743.

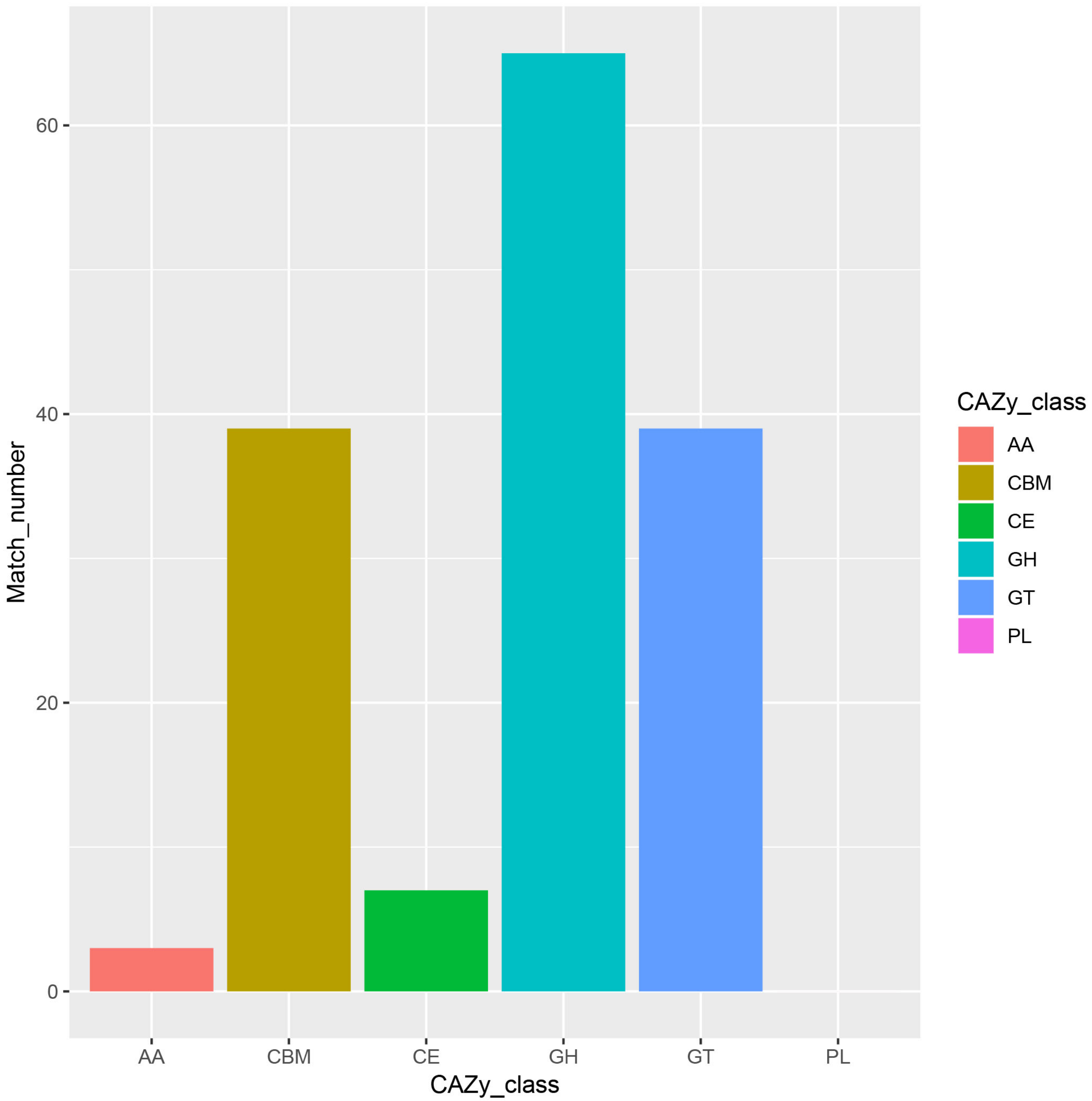

Fig. S2. CAZy annotation of the protein coding sequences of the genome of *V. vulnificus* strain MCCC 1A08743.

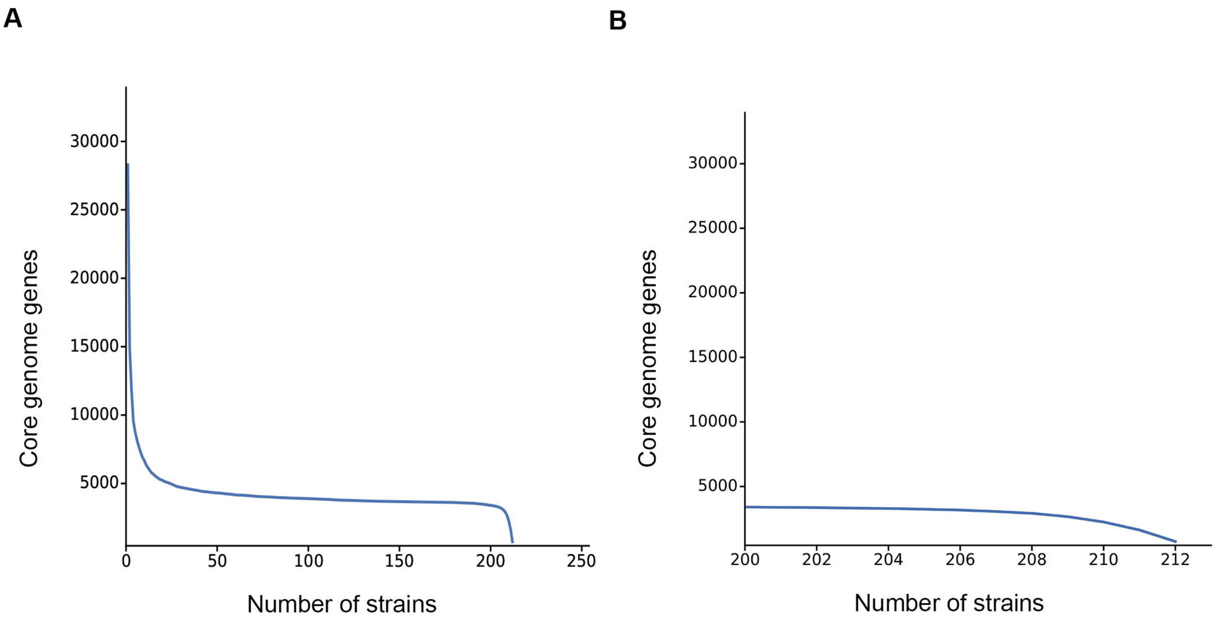

Fig. S3. ***V. vulnificus*** **core genome plot.** (A) Curve for core genome of 211 *V. vulnificus* strains. (B) Magnified view of the curve shown in A.

Table S1. Summary of gene annotation of *V. vulnificus* strain MCCC 1A08743.

Click here to download Table S1

Table S2. ANI value based on the entire genome sequence of 212 strains of *V. vulnificus.*

Click here to download Table S2

Table S3. Virulence factors annotation of *V. vulnificus* strain MCCC 1A08743. Information is based on data retrieved from the VFDB database.

Click here to download Table S3

Table S4. Characteristics of 211 strains of *V. vulnificus* retrieved from NCBI used in this study.

Click here to download Table S4
